# The Effects of Music Intervention on Functional Connectivity Strength of the Brain in Schizophrenia

**DOI:** 10.1155/2018/2821832

**Published:** 2018-05-02

**Authors:** Mi Yang, Hui He, Mingjun Duan, Xi Chen, Xin Chang, Yongxiu Lai, Jianfu Li, Tiejun Liu, Cheng Luo, Dezhong Yao

**Affiliations:** ^1^The Clinical Hospital of Chengdu Brain Science Institute, MOE Key Lab for Neuroinformation, University of Electronic Science and Technology of China, Chengdu, China; ^2^Department of Psychiatry, Chengdu Mental Health Centre, The Four People's Hospital of Chengdu, Chengdu 610036, China

## Abstract

Schizophrenia is often associated with behavior abnormality in the cognitive and affective domain. Music intervention is used as a complementary treatment for improving symptoms in patients with schizophrenia. However, the neurophysiological correlates of these remissions remain poorly understood. Here, we investigated the effects of music intervention in neural circuits through functional magnetic resonance imaging (fMRI) study in schizophrenic subjects. Under the standard care, patients were randomly assigned to music and non-music interventions (MTSZ, UMTSZ) for 1 month. Resting-state fMRI were acquired over three time points (baseline, 1 month, and 6 months later) in patients and analyzed using functional connectivity strength (FCS) and seed-based functional connection (FC) approaches. At baseline, compared with healthy controls, decreased FCS in the right middle temporal gyrus (MTG) was observed in patients. However, after music intervention, the functional circuitry of the right MTG, which was related with the function of emotion and sensorimotor, was improved in MTSZ. Furthermore, the FC increments were significantly correlated with the improvement of symptoms, while vanishing 6 months later. Together, these findings provided evidence that music intervention might positively modulate the functional connectivity of MTG in patients with schizophrenia; such changes might be associated with the observed therapeutic effects of music intervention on neurocognitive function. This trial is registered with ChiCTR-OPC-14005339.

## 1. Introduction

Schizophrenia is a psychiatric illness, which has been typically associated with complex and diverse impairments in cognitive, affective behavior and perceptual levels of processing, with a lifetime prevalence of 0.30% to 0.66% in the general population [1]. Schizophrenia may be characterized as a prototypical disorder of brain connectivity [2, 3]. The prevailing neuroimaging studies indicated that the dysfunction of the brain functional network maybe associated with the pathophysiological mechanisms underlying schizophrenia [2]. Antipsychotic drugs are commonly used prolonged treatments for schizophrenia. In addition, the complementary therapies, such as cognitive-behavioral therapy [4] and music intervention [5, 6], are also choices for patients with schizophrenia. Music intervention was known to significantly improve the psychiatric symptoms in schizophrenic subjects [6, 7]. There has been an interest in understanding the neural effects of music intervention, then gain a better understanding of the maintaining mechanisms of schizophrenia and improve further therapies.

Music is one of the oldest sociocognitive domains of humans. Music listening and music intervention (such as music performance and music listening by patients) are often documented by the proposed need for a medium for communication. Listening to Mozart K.448 could enhance spatial-temporal reasoning on subjects in the short-term [8]. Other forms of music were found to be equally temporarily effective [9, 10]. The findings of music intervention open a new page for the study of the effect of music on humans. To investigate the effect of music intervention on schizophrenic subjects, research has compared patients receiving standard care with or without music intervention [11]. Importantly, clinical reports have indicated that music intervention could have motivating, emotionally expressive, and relationship-building qualities in schizophrenia [12, 13]. After music intervention, significant advantages were detected in some measures concerning personal relations and subjectivity schizophrenic subjects [14]. Cognitive task performance could also be facilitated after listening to music by Mozart in patients with schizophrenia [15]. However, the particular mechanism behind this phenomenon is still poorly understood.

In the past decades, resting-state functional magnetic resonance imaging (fMRI), which measures ongoing spontaneous brain activity and maps interregional functional connectivity (FC) [16, 17], as a powerful in vivo imaging technique has received substantial attention in brain disorder researches [18, 19]. Our previous studies have demonstrated that altered brain FC of patients with schizophrenia was associated with psychiatric symptoms [20, 21]. Importantly, our previous study indicated that the insular cortex might be an important region in music intervention from a local aspect (seed-based FC analysis) for schizophrenic subjects [22]. However, the application of graph-based network analysis allows the computation of a wide range of measures, such as small-world attributes, hubs, and network modularity [23], which could characterize diverse topological properties of the brain disorder connectome from a global aspect. For patients with schizophrenia, reductions in topological measures (clustering coefficient, local efficiency) [24, 25] and small-world brain network [26] have been consistently found through fMRI. However, there were few studies from which correlates of music intervention in schizophrenic subjects from a global aspect have been investigated.

Here, we used fMRI to examine the effect of music intervention on schizophrenia. The distribution of high nodal connectivity in the brain plays vital roles in transferring information over the whole brain [27]. The hubs of the whole-brain network are generally affected in patients with schizophrenia based on the functional relevance and physiological basis [2]. Several studies have demonstrated that the functional connectivity strength (FCS) metric, which could examine hub connectivity through voxel-based graph analysis approaches, is closely associated with physiological measures such as regional cerebral blood flow, oxygen, and glucose metabolism [28]. Therefore, different from our previous work, in this study, fMRI was used to identify functional hubs of brain networks from the global aspect to assess the effect of music intervention on schizophrenia through computing the FCS at the voxel level. Such a voxel-wise approach enables whole brain hub mapping but overcomes the limitation of seed-based approaches for identifying and locating functional hubs in the brain. The goals of this study are to (1) explore the modulatory effect of music intervention on resting-state functional networks in patients with schizophrenia, (2) explore the relationship between music intervention-induced change of FC and change in psychiatric symptoms and neuropsychological in schizophrenia, and (3) assess the duration of effects of music intervention through studying the schizophrenic subjects six months later.

## 2. Material and Methods

### 2.1. Subjects

The participants in this study are the same with our previous study including fifty-six schizophrenic subjects (male: 10; female: 26) and nineteen healthy controls (HC) (male: 7; female: 12). All subjects were recruited from the clinical hospital of the Chengdu Brain Science Institute (CBSI). The detail fundamental information of schizophrenic subjects and HC could be referred to our previous article [22].

### 2.2. Design

To measure the effect of music intervention on schizophrenic subjects, a quasirandomized controlled trial was conducted in this study. Twenty-two patients with schizophrenia, who were randomly selected from patients, received group music intervention (MTSZ: music intervention patients with schizophrenia). Controlled patients (UMTSZ: non-music intervention patients with schizophrenia, remaining twenty-three patients) received standard care only. Three time point tests were included: a baseline test, a 1-month follow-up, and a 6-month follow-up. The blinded assessments and assignment were performed in schizophrenic subjects. The design of music intervention in this study was the same with our previous work. The detailed information could be referred from the previous article [22].

### 2.3. Content of Music Intervention

One professional music therapist participated in this study. Mozart's sonata K.448 music listening was performed in the MTSZ group (30 minutes per day, 30 days). MTSZ patients peacefully listened to the music in a quiet room during each session. The music therapist introduced the background of the music to schizophrenic patients at the beginning. The UMTSZ was treated solely with antipsychotic drugs. The content of the music intervention was also the same with our previous study. The detailed information could be referred from the previous article [22].

### 2.4. Psychiatric and Neuropsychological Assessment

Three evaluators of the neuropsychological assessments and one psychiatrist assessed the patients with schizophrenia. The psychiatric symptoms of the schizophrenic patients were assessed by a psychiatrist using the Positive and Negative Symptom Scale (PANSS). In the neuropsychological assessment (Block Design Test (BDT), BVRT, and Spatial Maze Test), the evaluators were trained to achieve a high interrater reliability before the start of the study. Detailed information can be found in Section 1 of the Supplemental Information).

### 2.5. Data Acquisition

Experiments were performed on a 3 T MRI scanner (GE Discovery MR750). A detailed description of the data collection can be found in Section 2 of the Supplemental Information. Functional image preprocessing was performed using SPM8 (Statistical Parametric Mapping, http://www.fil.ion.ucl.ac.uk/spm/) according to a standard pipeline. Slice timing correction and head motion correction were carried out. Then, the functional scans were normalized to the Montreal Neurological Institute (MNI) EPI template and resampled to 3 × 3 × 3 mm^3^ voxels. The general processing procedures of spatial smoothing (Gaussian kernel of a full width at half maximum (FWHM) of 6 mm) was not included for the FCS analysis but was included for the FC analysis. Then, nuisance signals were then removed from the data through linear regression (white matter (WM), cerebrospinal fluid (CSF), and six motion parameters), except the global signal due to recent excellent studies [29]. Finally, fMRI data were temporally filtered in band-pass 0.01–0.08 Hz. Recent studies demonstrated that head motion had a substantial impact on FC [30, 31]. Thus, any subjects who had a maximum translation in any of the cardinal directions larger than 2.0 mm or a maximum rotation larger than 2.0 degrees were excluded. Besides, we also assessed framewise displacement (FD) in three groups as suggested by Power et al. [30] using the following formula:

(1) $$\begin{array}{c} FD=\frac{1}{M-1}\overset{M}{\underset{i=2}{\sum }}\sqrt{{\left|\Delta t_{x_{i}}\right|}^{2}+{\left|\Delta t_{y_{i}}\right|}^{2}+{\left|\Delta t_{z_{i}}\right|}^{2}+{\left|\Delta d_{x_{i}}\right|}^{2}+{\left|\Delta d_{y_{i}}\right|}^{2}+{\left|\Delta d_{z_{i}}\right|}^{2}}, \end{array}$$

where *M* is the length of the time courses (*M* = 250 in this study); *x*_*i*_, *y*_*i*_, and *z*_*i*_ are translations/rotations at the *i*th time point in the x, y, and z directions, respectively; Δ*t* represents the framewise displacement translation; and Δ*d* represents the framewise displacement rotation. Δ*d*_*x*_*i*__ = *x*_*i*_ − *x*_*i*−1_ and similar for *d*_*y*_*i*__and*d*_*z*_*i*__.

Structural images were processed using SPM8 toolbox. Spatial normalization to MNI-space was performed using a diffeomorphic anatomical registration through exponentiated lie algebra and segmented into gray matter (GM), WM, and CSF. The segmented GM and WM were modulated using nonlinear deformation. Total Intracranial Volume (TIV) and GM volume and were calculated. Then, the normalization analysis was performed: the GM volume was divided by the TIV score. The schizophrenic patients' and HC's normalized GM at baseline were entered as a global variable to correct for the global GM volume of different subjects in the statistical analysis.

### 2.6. Functional Connectivity Strength Analysis

Whole-brain FC analysis was performed as follows. First, to exclude artefactual correlations from non-gray matter, the GM mask was generated by thresholding (cutoff = 0.2) the average of GM probability map involving all subjects. The time series from each voxel within the GM mask was extracted, and Pearson's correlations between any pair of voxels were calculated for each subject. To improve normality, we then transformed individual correlation matrices to a *z*-score matrix using a Fisher *r*-to-*z* transformation. Then, for a given voxel (node), nodal FCS was computed as the sum of weights of its connections with other voxels. We conservatively restricted our analysis to positive correlations above the threshold of *r* = 0.2, which was chosen to eliminate the voxels with weak correlations attributable to signal noise [32]. Before group-level statistical analysis, an individual voxel-wise FCS map was standardized to *z*-scores and further spatially smoothed (FWHM = 6 mm).

### 2.7. Functional Connectivity Analysis

We examined FC by taking the brain region that had showed different FCS (pursuant to the FCS statistical test) as the seed. FC analysis was performed between the seed and all voxels in the brain. To improve normality, we then transformed individual correlation matrices to a *z*-score matrix using a Fisher *r*-to-*z* transformation. In this manner, FC maps of these seeds were produced for each subject.

### 2.8. Statistical Analysis

#### 2.8.1. Participant Fundamental Information Statistics

Age, years of education, and FD among the three groups were compared using one-way ANOVA. Chi-square test was used to compare gender distributions. A two-sample *t*-test was used to compare the psychiatric symptoms (illness duration, medication dosage in chlorpromazine (CPZ) equivalents (mg), and PANSS scores) between two groups of patients at baseline. Repeated measure ANOVA were performed to determine the music intervention∗time interaction, main effects of music intervention and time on neuropsychological measurements, and PANSS scores.

#### 2.8.2. Baseline Brain Connectivity Analyses between Patients and HC

Firstly, we established baseline abnormalities between HCs and schizophrenic patients through two-sample *t*-test in FCS and seed-based FC, respectively, with age, gender, years of education, and GM as covariates. The comparison, for the FCS statistical test, was constrained within the GM mask. The seed-based FC statistical test was performed within the mask that resulted from the union set of the one-sample *t*-test of the two groups (*p* < 0.005 with cluster-level false discovery rate corrected, *p* < 0.05). Due to the quantity of patients being greater than the amount of HC, the HC and the same number of patients who were randomly selected from the whole sample of patients were entered into the statistical comparisons. These steps were repeatedly performed 200 times [33]. A total of 200 comparison results were obtained. Then, we calculated the probability map where the voxels exhibited significant differences (*p* < 0.005 with cluster-level false discovery rate corrected, *p* < 0.05) across the 200 comparisons.

Furthermore, we also established the difference between MTSZ and UMTSZ at baseline through a two-sample *t*-test in FCS and seed-based FC, respectively, with gender, years of education, GM, and age as covariates. The significance threshold of the group difference was set to *p* < 0.05.

#### 2.8.3. Longitudinal Analysis of Functional Connectivity in Patients

After tests of normality, homogeneity of variance, and Mauchly's test of sphericity, the repeated-measure ANOVA and post hoc analyses were performed on the schizophrenic subjects from baseline and 1-month follow-up to determine the music intervention∗time interaction, main effects of music intervention, and time on FCS and seed-based FC, respectively. The age, gender, illness duration, education characteristics, GM, and the medication dosage were used as covariates. The significance threshold for ANOVA was set to *p* < 0.005 with cluster-level false discovery rate corrected (*p* < 0.05). For the region with difference resulted from repeated-measure ANOVA, a further two-sample *t*-test was performed between HC and patients (MTSZ and UMTSZ from 1-month follow-up) to assess the effect of the 1-month music intervention on patients relative to HC. At last, the data with the 6-month follow-up was used to evaluate the long-term effect of music intervention through the comparison to data from the 1-month follow-up in schizophrenic subjects.

### 2.9. The Relationship between FC and Patients' Variables

To investigate the underlying relationship between the changes of functional measurements (i.e., FCS and seed-based FC) and the changes of neuropsychological measurements, as well as PANSS in the MTSZ and UMTSZ groups, we used partial correlation analysis in this study. We extracted the mean *z*-score of the region that showed significant difference of FCS and FC. Then, the partial correlations between the mean changes (1-month later minus baseline) and the percentage change scores (1 − [score at 1-month/score at baseline]) of neuropsychological measurements were performed, as well as the percentage change scores of PANSS, with age, gender, illness duration, education characteristics, GM, and medication dosage as covariates. The partial correlation analysis was also performed between the mean changes (6 months later minus baseline) and the percentage change scores (1 − [score at 6 months/score at baseline]) of neuropsychological and PANSS scores.

## 3. Results

### 3.1. Participant Demographic Information

Forty-five patients with schizophrenia finished the randomized controlled trial at two time points (baseline and 1-month follow-up). Nine of them (4 in MTSZ and 5 in UMTSZ) were excluded due to excessive head motion. Thus, 18 MTSZ, 18 UMTSZ, and 19 HC were included in the following analysis.

Furthermore, at 6-month follow-up, 9 schizophrenic subjects (25%) were not recalled because they were no longer interested in the experiment after being discharged from the hospital. 5 patients with schizophrenia with excessive head motion were also excluded, so thirteen (13/18) UMTSZ and nine (9/18) MTSZ patients were included.

### 3.2. Changes in Psychiatric Symptoms and Neuropsychological Measurements

We observed significant music intervention main effect and music intervention∗time interaction on the scores of Positive and Negative Symptom Scale (PANSS) and the score of Benton Visual Retention Test (BVRT). Post hoc analysis revealed that significant increase of BVRT and decrease of PANSS scores were found in MTSZ at 1-month follow-up, while not in the UMTSZ group. Nonsignificant difference was observed between the MTSZ and UMTSZ groups at baseline. Furthermore, the effects of music intervention had vanished in the MTSZ at the 6-month follow-up (comparison results between the schizophrenic subjects from 1-month follow-up and 6-month follow-up). The detailed information could be referred from the previous study [22].

### 3.3. The Abnormalities of FCS at Baseline

**Figure 1:**
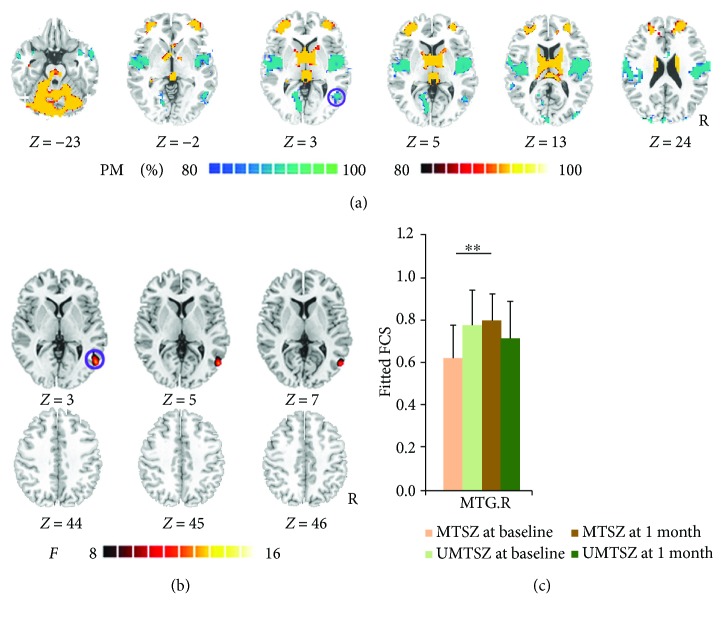
Music intervention∗time interaction on functional connectivity strength (FCS). (a) denotes the altered FCS in patients compared with healthy controls at baseline. The cool color indicates decreased functional connections, and the hot color indicates increased functional connections. All of the maps are shown with a probability score between 80% and 100%. (b) denotes that significant music intervention∗time interaction on FCS was observed in the right middle temporal gyrus (MTG.R) through repeated-measure ANOVA analysis. (c) denotes the post hoc analysis results in regions showing significant music intervention∗time interaction on FCS. The data were expressed as the mean value + standard error. ^∗∗^ *p* < 0.01. The violet circles mark the same region in (a) and (b), which means the positively modulated region through music intervention in patients.

Firstly, the FCS patterns were remarkably similar across the MTSZ and UMTSZ, as well as the HC (see Supplementary Figures S1 and S2). The FCS patterns were similar to those observed in previous studies [28]. At baseline, compared to HC, in patients with schizophrenia, the decreased FCS was observed in the posterior insula, prepostcentral gyrus, supplementary motor area, middle cingulate cortex (MCC), and middle temporal gyrus (MTG) (Figure 1(a)). The patients with schizophrenia also exhibited increased FCS in the basal ganglia, bilateral middle frontal gyrus, and cerebellum. These findings are similar with previous researches [26]. No significant difference FCS was observed between MTSZ and UMTSZ at baseline.

### 3.4. Longitudinal Changes of FCS after Music Intervention in Schizophrenia

In the FCS analysis, significant music intervention∗time interaction on FCS was observed in the right MTG (Figure 1(b), Table 1). Post hoc analysis revealed that significantly increased FCS was observed in MTSZ following 1-month music intervention (right MTG: *t*_17_ = 2.97, *p* = 0.008) (Figure 1(c)), while no difference was found in the UMTSZ group. FCS in these regions did not show any significant difference between the two patient groups at baseline.

Additionally, following the 1-month music intervention, significantly decreased FCS was observed in the right MTG in MTSZ (*t*_35_ = −2.24, *p* = 0.031) and UMTSZ (*t*_35_ = −2.48, *p* = 0.018) compared with HC.

Finally, in MTSZ, the FCS of the right MTG 6 months later had a significant decrease compared with the data from the 1-month follow-up. In a word, a diminished effect of music intervention was observed at the 6-month follow-up.

### 3.5. Longitudinal Changes of FC after Music Intervention in Schizophrenia

**Figure 2:**
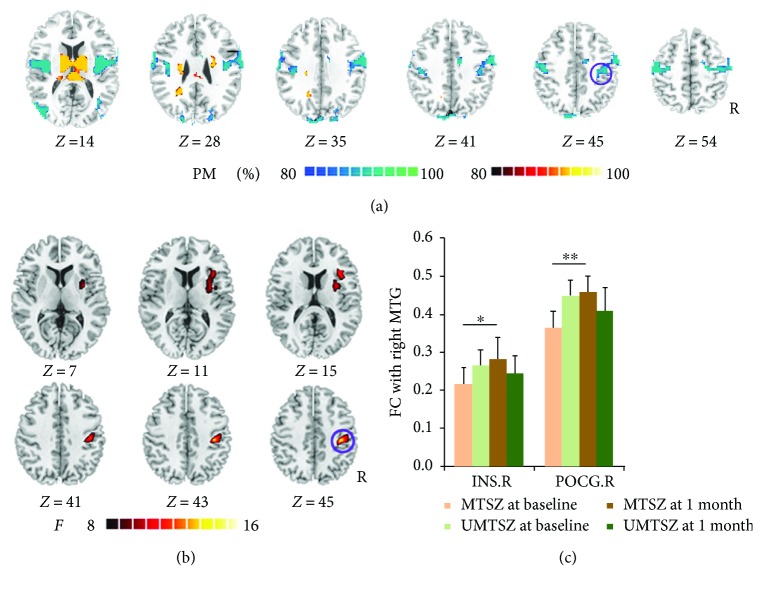
Music intervention∗time interaction on FC of the right middle temporal gyrus (MTG.R). (a) denotes that the altered FC resulted from seeded-MTG.R FC analysis in patients compared with healthy controls at baseline. The cool color indicates decreased functional connections, and the hot color indicates increased functional connections. All of the maps are shown with a probability score between 80% and 100%. (b) denotes that significant music intervention∗time interaction on FC was observed between the right MTG and right precentral gyrus (POCG.R), as well as the right insula (INS.R). (c) The bar maps present the between-group and within-group differences in regions showing significant music intervention∗time interaction on FC of the MTG.R. The data were expressed as the mean value + standard error. ^∗^ *p* < 0.05, ^∗∗^ *p* < 0.01. The violet circles mark the same region in (a) and (b), which means the positively modulated region through music therapy in patients.

Based on the FCS results in MTSZ, we assessed the longitudinal changes of resting-state FC of the right MTG in the patient groups. First, at baseline, the patients exhibited declined MTG FCs with the insula, pre-/postcentral gyrus, and superior occipital gyrus. Increased FCs were also observed between the MTG and cerebellum (Figure 2(a)).

Second, ANOVA analysis showed that significant music intervention∗time (1 month) interaction on the FC of the right MTG was located in the right anterior insula and postcentral gyrus (Figure 2(b) and Table 2). Post hoc analysis showed significant increases in MTSZ following 1-month music intervention (right anterior insula: *t*_34_ = 2.55, *p* = 0.020; right postcentral: *t*_34_ = 3.19, *p* = 0.005) compared to baseline (Figure 2(c)) but no significance in UMTSZ.

Third, compared with HC, both patient groups no significant changes were found between the MTG and right anterior insula and postcentral gyrus, except for the FC between the MTG and right postcentral gyrus in UMTSZ (*t*_35_ = −2.27, *p* = 0.029).

**Figure 3:**
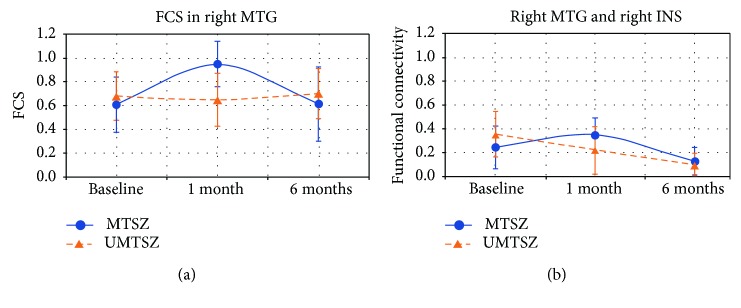
6-month effects of music intervention versus nonmusic intervention on brain functional connectivity strength (FCS) and functional connectivity (FC) in patients with schizophrenia. The data were expressed as the mean value ± standard error. (a) denotes the long-term effects of music intervention versus nonmusic intervention on FCS of the middle temporal gyrus (MTG) in patients with schizophrenia. (b) denotes the long-term effects of music intervention on FC between the MTG and right insula (INS).

The 6-month follow-up investigation is illustrated in Figure 3. In MTSZ, the FC between the MTG and insula at 6 months later had a significant decrease compared with the data from the 1-month follow-up. In a word, a diminished effect of music intervention was observed at the 6-month follow-up.

### 3.6. The Relationship between Altered FC and Patients' Variables

**Figure 4:**
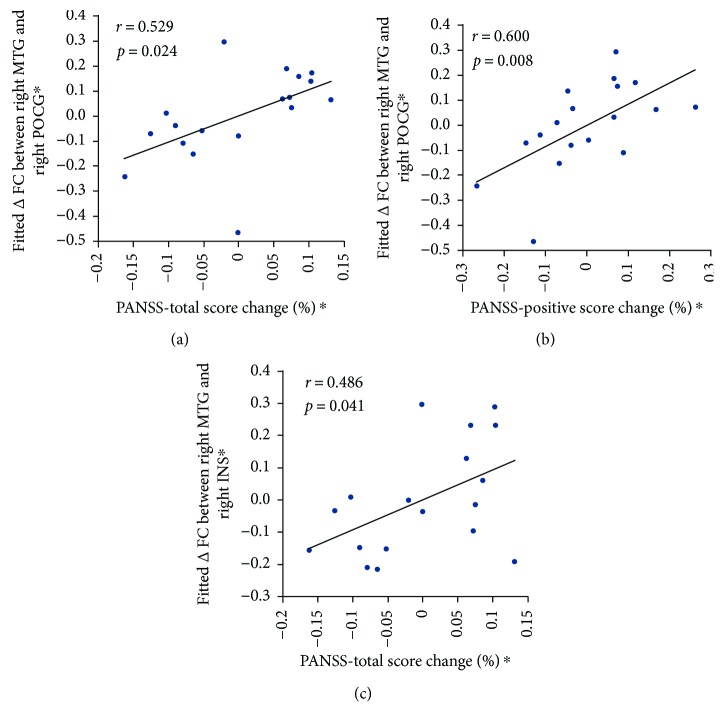
The relationship between altered functional connectivity (FC) and patient's scores of PANSS. (a) and (b) denote the significant correlations between the FC change (right MTG and right postcentral) and the percentage change scores of PANSS-total score (a) and PANSS-positive score (b). (c) denotes the significant relationship between the FC change (right MTG and right insula) and the percentage change score of PANSS-total score. Δ = week 4 − baseline. ∗ means the residual value after the regression analysis.

In MTSZ, partial correlation analysis revealed that MTG-postcentral FC change showed a significant positive correlation with the change scores of PANSS-total score, as well as PANSS-positive score (Figure 4(a): PANSS-total score: *r* = 0.529, *p* = 0.024; Figure 4(b): PANSS-positive score: *r* = 0.600, *p* = 0.008). Similar associations result with the change score of PANSS-total score, which was also found in the changes FC (MTS and anterior insula) (Figure 4(c): *r* = 0.486, *p* = 0.041). No significant correlations were found between the changes in brain functional measures (FCS and FC) in the areas and the change score of neuropsychological measurements, as well as medication dosage in MTSZ and UMTSZ. There were no significant relationships in UMTSZ. Finally, the significant relationship was also not observed between the changes of functional measures (6 months later minus baseline) and changes of neuropsychological score, as well as the PANSS score (6 months later minus baseline) in MTSZ and UMTSZ.

## 4. Discussion

This study combined resting-state fMRI and voxel-based graph approach to characterize the effect of music intervention on the brain network hub and FC changes in schizophrenic subjects. Our findings revealed that the 1-month music intervention had the positive improving effect on the abnormally lower hub score of the right MTG in patients. Furthermore, we found that the intervention-related resting-state FC was also being positively modulated in the right MTG functional network. Finally, the psychiatric symptom analysis indicated that the changes of FCs showed significant positive relationship with the change scores of PANSS, while vanishing 6 months later. These findings provide evidence supporting the notion that music intervention might positively improve the FC between the MTG and insula, as well as the sensorimotor gyrus. These changes may be related to the remission of psychiatric symptoms in schizophrenia.

While disturbances in higher order brain functions, such as memory [34] and cognitive [35], are well known in schizophrenia, recent studies have also documented basic sensory-processing deficits. In schizophrenic subjects, perceptual deficits have become increasingly observed in the visual system [36–38]. The early visual system is divided into two basic subdivisions, including parvocellular and magnocellular divisions. The magnocellular neurons are projected to the dorsal visual stream, such as the MTG and intraparietal sulcus, which process visual information and guide action [39, 40]. There are additional projections through the thalamus to subcortical regions, which may play a specific role in emotional expression [40, 41]. Previous excellent research reported that music could be associated with increased functional coupling between emotional and attentional brain regions in the parietal and visual regions (MTG) in patients with visual neglect [42]. In this study, we observed the decreased FCS in the right MTG in schizophrenic subjects at the baseline, while we found music intervention could positively improve the abnormally lower FCS score of the right MTG. Our finding might be indicated that MTG may be a target gyrus in the dorsal visual stream for music intervention. Moreover, the altered FCS of MTG might positively regulate the processing of differentially sensitive motion to emotion in schizophrenia.

The deficits of the visual magnocellular pathway appear to be related to higher level emotional and cognitive impairments in schizophrenia [43–45]. Recent study also reported that the insular cortex and striatum are highly associated with visual magnocellular regions, which may serve as the neuroanatomical substrate in the perception of emotion [41]. Through music intervention, we found increased FC between the right MTG and the right anterior insula which has been thought to have a role in tracking emotions [46, 47]. Increased functional integration between the MTG and insular cortex might have an improving effect on emotional processing in schizophrenic subjects. The abovementioned finding might reflect that the music intervention increased functional integration between the MTG and insula. The changed FC might improve higher order processes such as emotional identification and experience in MTSZ. In addition, the significant relationship was also observed between the changed FC of the insula and the change score of PANSS-total. We propose that the music intervention might positively modulate the psychiatric symptom of schizophrenic subjects.

Reduced activation region in the visual magnocellular pathway was related to the deficits in motion processing in schizophrenia [48]. Our previous study revealed that music training could increase FC in the motor, visual, and multisensory cortices of musicians [49]. In the present study, the right MTG was observed to have increased FC with the right postcentral gyrus in MTSZ following the 1-month music intervention. The postcentral gyrus has been reported as a functional plasticity region in the musician through musical training [49]. In addition, increased BVRT scores, which was a well-established neurodiagnostic instrument to assess visuospatial [50, 51], were also observed though music intervention. Furthermore, the psychiatric symptom analysis revealed that the significant relationships were observed between the changed FC of the postcentral gyrus and the change scores of PANSS in MTSZ. These findings might reflect that the music intervention increased functional integration between visual and sensorimotor networks, which might improve the processing of visual information and guide action in schizophrenic subjects.

While we believe our findings provide new insight into the role of brain FC in understanding the effects of music intervention, several limitations need to be further addressed. First, a single musical piece was selected (Mozart's sonata K.448) in the present study. The special effects of Mozart's music might be observed in patients. Further studies should investigate the effects of other types of music, such as general, familiar, and preferred music, in schizophrenic subjects. The future research might be a better way to understand the effect of different types of music on patients as well as to investigate whether these effects are similar to those obtained from Mozart music. Second, the correlation coefficient threshold, which is to eliminate weak correlations possibly arising from noise signal, is not unique during the FCS analysis, and this fixed value may lead to some false positive or negative findings. The threshold value of 0.2 was used in this study based on prior knowledge. We also performed the same processing using two other thresholds (i.e., 0.1 and 0.3); these results were similar. Finally, the results of this study require replication in larger sample size studies.

## 5. Conclusions

This study demonstrated that music intervention might positively improve the functional hub of the MTG within the visual magnocellular pathway and simultaneously lead to the change in FC with some other regions related with function of emotion and sensorimotor within the brain circuitry of schizophrenic subjects. Furthermore, we observed related modulation in psychiatric symptoms and neuropsychological measurements in schizophrenia following the music intervention. Specifically, these positive modulations vanished 6 months later. These findings provided new insights into the effects of music intervention in medicine at the level of FC and might lead to treatment strategies including sensory-processing rehabilitation through music intervention.

## Figures and Tables

**Table 1:** Significant music intervention∗time interaction on FCS of the whole brain through repeated-measure ANOVA.

Regions	BA	MNI coordinates			Peak *F*-score	Cluster voxels
		*x*	*y*	*z*		
MTG.R	BA 37	52	−64	3	12.632	103

BA: Brodmann area; MTG: middle temporal gyrus.

**Table 2:** Significant music intervention∗time interaction on FC of the middle temporal gyrus through repeated-measure ANOVA.

Regions	BA	MNI coordinates			Peak *F*-score	Cluster voxels
		*x*	*y*	*z*		
POCG.R	BA 3	40	−25	46	14.319	123
INS.R	BA 48	36	16	15	9.108	101

BA: Brodmann area; POCG: postcentral gyrus; INS: insula.

## Supplementary Materials

Supplementary Materials Figure S1: functional connectivity strength (FCS) maps. Mean FCS maps in the MTSZ and UMTSZ groups at baseline and 1 month. The color bar represents the strength of FCS. Figure S2: functional connectivity strength (FCS) maps. Mean FCS maps in the healthy controls (HC). The color bar represents the strength of FCS.

## References

[1] McGrath J., Saha S., Chant D., Welham J. (2008). Schizophrenia: a concise overview of incidence, prevalence, and mortality.

[2] Fornito A., Zalesky A., Pantelis C., Bullmore E. T. (2012). Schizophrenia, neuroimaging and connectomics.

[3] Kambeitz J., Kambeitz-Ilankovic L., Leucht S. (2015). Detecting neuroimaging biomarkers for schizophrenia: a meta-analysis of multivariate pattern recognition studies.

[4] Rector N. A., Beck A. T. (2001). Cognitive behavioral therapy for schizophrenia: an empirical review.

[5] Shih Y. N., Chen C. S., Chiang H. Y., Liu C. H. (2015). Influence of background music on work attention in clients with chronic schizophrenia.

[6] Lu S. F., Lo C. H. K., Sung H. C., Hsieh T. C., Yu S. C., Chang S. C. (2013). Effects of group music intervention on psychiatric symptoms and depression in patient with schizophrenia.

[7] Peng S.-M., Koo M., Kuo J.-C. (2010). Effect of group music activity as an adjunctive therapy on psychotic symptoms in patients with acute schizophrenia.

[8] Rauscher F. H., Shaw G. L., Ky C. N. (1993). Music and spatial task performance.

[9] Rauscher F., Shaw G., Levine L., Wright E., Dennis W., Newcomb R. (1997). Music training causes long-term enhancement of preschool children’s spatial–temporal reasoning.

[10] Gardiner M. F., Fox A., Knowles F., Jeffrey D. (1996). Learning improved by arts training.

[11] Ulrich G., Houtmans T., Gold C. (2007). The additional therapeutic effect of group music therapy for schizophrenic patients: a randomized study.

[12] Solli H. P. (2008). “Shut up and play!” improvisational use of popular music for a man with schizophrenia.

[13] Rolvsjord R. (2001). Sophie learns to play her songs of tears.

[14] Hayashi N., Tanabe Y., Nakagawa S. (2002). Effects of group musical therapy on inpatients with chronic psychoses: a controlled study.

[15] Glicksohn J., Cohen Y. (2000). Can music alleviate cognitive dysfunction in schizophrenia?.

[16] Biswal B., Zerrin Yetkin F., Haughton V. M., Hyde J. S. (1995). Functional connectivity in the motor cortex of resting human brain using echo-planar MRI.

[17] Fox M. D., Raichle M. E. (2007). Spontaneous fluctuations in brain activity observed with functional magnetic resonance imaging.

[18] Luo C., Li Q., Lai Y. (2011). Altered functional connectivity in default mode network in absence epilepsy: a resting-state fMRI study.

[19] Dong L., Luo C., Zhu Y. (2016). Complex discharge-affecting networks in juvenile myoclonic epilepsy: a simultaneous EEG-fMRI study.

[20] Chen X., Duan M., Xie Q. (2015). Functional disconnection between the visual cortex and the sensorimotor cortex suggests a potential mechanism for self-disorder in schizophrenia.

[21] Duan M., Chen X., He H. (2015). Altered basal ganglia network integration in schizophrenia.

[22] He H., Yang M., Duan M. (2018). Music intervention leads to increased insular connectivity and improved clinical symptoms in schizophrenia.

[23] Bullmore E., Sporns O. (2009). Complex brain networks: graph theoretical analysis of structural and functional systems.

[24] Liu Y., Liang M., Zhou Y. (2008). Disrupted small-world networks in schizophrenia.

[25] Alexander-Bloch A. F., Gogtay N., Meunier D. (2010). Disrupted modularity and local connectivity of brain functional networks in childhood-onset schizophrenia.

[26] Lynall M. E., Bassett D. S., Kerwin R. (2010). Functional connectivity and brain networks in schizophrenia.

[27] Achard S., Salvador R., Whitcher B., Suckling J., Bullmore E. (2006). A resilient, low-frequency, small-world human brain functional network with highly connected association cortical hubs.

[28] Liang X., Zou Q., He Y., Yang Y. (2013). Coupling of functional connectivity and regional cerebral blood flow reveals a physiological basis for network hubs of the human brain.

[29] Yang G. J., Murray J. D., Repovs G. (2014). Altered global brain signal in schizophrenia.

[30] Power J. D., Barnes K. A., Snyder A. Z., Schlaggar B. L., Petersen S. E. (2012). Spurious but systematic correlations in functional connectivity MRI networks arise from subject motion.

[31] Satterthwaite T. D., Wolf D. H., Loughead J. (2012). Impact of in-scanner head motion on multiple measures of functional connectivity: relevance for studies of neurodevelopment in youth.

[32] Wang L., Xia M., Li K. (2015). The effects of antidepressant treatment on resting-state functional brain networks in patients with major depressive disorder.

[33] Li H.-J., Xu Y., Zhang K.-R., Hoptman M. J., Zuo X.-N. (2015). Homotopic connectivity in drug-naïve, first-episode, early-onset schizophrenia.

[34] Achim A. M., Bertrand M. C., Sutton H. (2007). Selective abnormal modulation of hippocampal activity during memory formation in first-episode psychosis.

[35] Weinberger D. R., Gallhofer B. (1997). Cognitive function in schizophrenia.

[36] Li C.-S. R. (2002). Impaired detection of visual motion in schizophrenia patients.

[37] Schechter I., Butler P. D., Silipo G., Zemon V., Javitt D. C. (2003). Magnocellular and parvocellular contributions to backward masking dysfunction in schizophrenia.

[38] Martinez A., Hillyard S. A., Dias E. C. (2008). Magnocellular pathway impairment in schizophrenia: evidence from functional magnetic resonance imaging.

[39] Butler P. D., Javitt D. C. (2005). Early-stage visual processing deficits in schizophrenia.

[40] Bedwell J. S., Chan C. C., Cohen O., Karbi Y., Shamir E., Rassovsky Y. (2013). The magnocellular visual pathway and facial emotion misattribution errors in schizophrenia.

[41] Vuilleumier P., Armony J. L., Driver J., Dolan R. J. (2003). Distinct spatial frequency sensitivities for processing faces and emotional expressions.

[42] Soto D., Funes M. J., Guzman-Garcia A., Warbrick T., Rotshtein P., Humphreys G. W. (2009). Pleasant music overcomes the loss of awareness in patients with visual neglect.

[43] Brenner C. A., Lysaker P. H., Wilt M. A., O'Donnell B. F. (2002). Visual processing and neuropsychological function in schizophrenia and schizoaffective disorder.

[44] Sergi M. J., Rassovsky Y., Nuechterlein K. H., Green M. F. (2006). Social perception as a mediator of the influence of early visual processing on functional status in schizophrenia.

[45] Butler P. D., Abeles I. Y., Weiskopf N. G. (2009). Sensory contributions to impaired emotion processing in schizophrenia.

[46] Uddin L. Q. (2015). Salience processing and insular cortical function and dysfunction.

[47] Gray M. A., Critchley H. D. (2007). Interoceptive basis to craving.

[48] Kim D., Wylie G., Pasternak R., Butler P. D., Javitt D. C. (2006). Magnocellular contributions to impaired motion processing in schizophrenia.

[49] Luo C., Guo Z. W., Lai Y. X. (2012). Musical training induces functional plasticity in perceptual and motor networks: insights from resting-state FMRI.

[50] Strauss E., Sherman E. M., Spreen O. (2006).

[51] Tamkin A. S., Kunce J. T. (1985). A comparison of three neuropsychological tests: the Weigl, Hooper, and Benton.

