# Supplementary material for: The Effects of Music Intervention on Functional Connectivity Strength of the Brain in Schizophrenia
Source: Neural Plast. 2018 May 2;2018:2821832. doi: 10.1155/2018/2821832 (PMC5954893; doi:10.1155/2018/2821832)
Supplement: Supplementary Materials — Figure S1: functional connectivity strength (FCS) maps. Mean FCS maps in the MTSZ and UMTSZ groups at baseline and 1 month. The color bar represents the strength of FCS. Figure S2: functional connectivity strength (FCS) maps. Mean FCS maps in the healthy controls (HC). The color bar represents the strength of FCS. [file 2821832.f1.pdf]

The Effects of Music intervention on Functional connectivity strength of Brain in Schizophrenia

Supplemental Information

Mi Yang[1,2#], Hui He[1#], Mingjun Duan[1,2], Xi Chen[1], Xin Chang[1], Yongxiu Lai[1], Jianfu Li[1], Tiejun Liu[1]*, Cheng Luo[1]*, Dezhong Yao[1]

1, The Clinical Hospital of Chengdu Brain Science Institute, MOE Key Lab for Neuroinformation, University of Electronic Science and Technology of China, Chengdu, China

2, Department of Psychiatry, Chengdu Mental Health Centre, The Four People's Hospital of Chengdu, Chengdu, 610036, China

*Address correspondence to: Cheng Luo, No. 4, Section 2, North Jianshe Road, Chengdu, P.R. China, 610054. E-mail: chengluo@uestc.edu.cn; Tel: 86 28 61831272; Fax: 86 28 83206978 Tiejun Liu, No. 4, Section 2, North Jianshe Road, Chengdu, P.R. China, 610054. E-mail: liutiejun@uestc.edu.cn; Tel: 86 28 83201018; Fax: 86 28 83206978.

[#]MY and HH contributed to this work equally

Section1. Neuropsychological measurements

Moreover, we administered Block Design Test (BDT), the Benton Visual Retention Test (BVRT), as well as Spatial Maze Test from the Wechsler Adult Intelligence Scale Revised (WAIS-R) [1] at three timepoints (baseline, 1-month later and 6-month later) during the music intervention in the two patient groups. The BDT, which reflect the visuospatial ability [2], requires the subjects to duplicate 10 target patterns using a set of two-colored blocks. The patterns were presented in order of ascending difficulty. The Benton Visual Retention Test is a well-established neurodiagnostic instrument that had been used to assess visuospatial perception and retention [3, 4]. The target patterns containing geometric and abstract figures were displayed to the subjects for 10 seconds. After that, the subjects were required to duplicate the figures from immediate memory. Finally, the Spatial Maze, which also comes from the Wechsler Adult Intelligence Scale, is one of the most reliable measures of visuospatial anterograde memory function.

Section2. Imaging data acquisition

Experiments were performed on a 3T MRI scanner (GE DISCOVERY MR750) in the University of Electronic Science and Technology of China. During scanning, we used foam padding and ear plugs to reduce head motion and scanning noise, respectively. Resting state functional MRI data were acquired using gradient-echo echo planar imaging sequences (repetition time [TR] = 2000 ms, echo time [TE] = 30 ms, flip angle [FA] = 90°, matrix = 64 × 64, field of view [FOV] = 24 × 24 cm^2, slice thickness/gap = 4 mm/0.4 mm), with an eight channel-phased array head coil. All subjects underwent a 510-second resting state scan to yield 255 volumes (32 slices per volume). The first five volumes were discarded for the magnetization equilibrium. Subsequently, High-resolution T1-weighted images were acquired using a 3-dimensional fast spoiled gradient echo sequence (TR = 6.008 msec, FA = 9°, matrix = 256 × 256, FOV = 25.6 × 25.6 cm^2, slice thickness = 1 mm, no gap, 152 slices). During resting-state fMRI, all subjects were instructed to have their eyes-closed and to move as little as possible without falling asleep.

Fig.S1

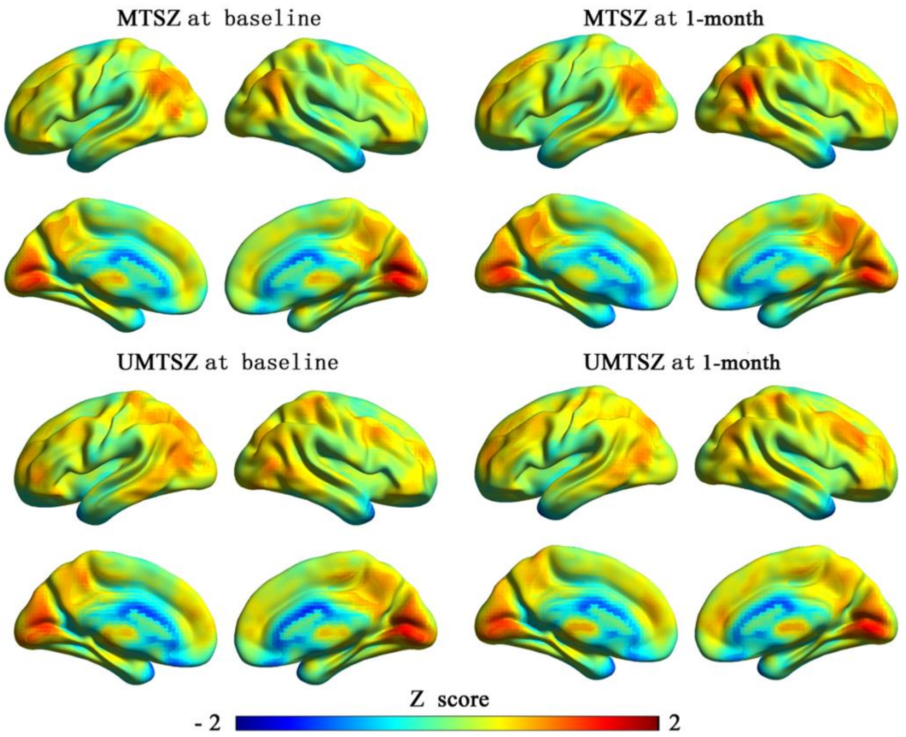

Fig.S1. Functional connectivity strength (FCS) maps. Mean FCS maps in the MTSZ and UMTSZ groups at baseline and 1-month. The color bar represents the strength of FCS.

Fig.S2

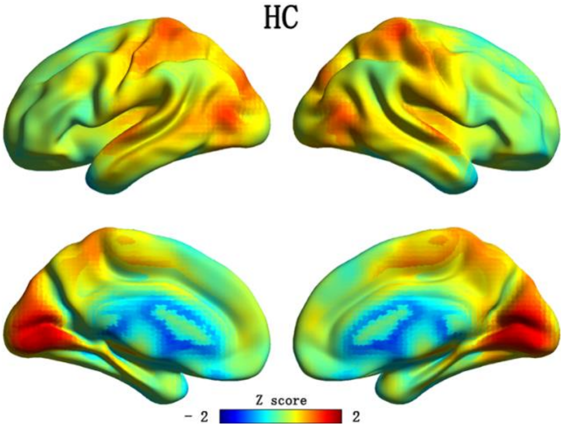

Fig.S2 Functional connectivity strength (FCS) maps. Mean FCS maps in the healthy controls (HC). The color bar represents the strength of FCS.

References:

1. Wechsler, D., *WAIS-R manual: Wechsler adult intelligence scale-revised*. 1981: Psychological Corporation.
2. Kaufman, A.S., *WAIS-III IQs, Horn's theory, and generational changes from young adulthood to old age.* Intelligence, 2001. **29**(2): p. 131-167.
3. Strauss, E., E.M. Sherman, and O. Spreen, *A compendium of neuropsychological tests: Administration, norms, and commentary*. 2006: Oxford University Press, USA.
4. Tamkin, A.S. and J.T. Kunce, *A comparison of three neuropsychological tests: The Weigl, Hooper, and Benton.* Journal of clinical psychology, 1985. **41**(5): p. 660-664.
